# Broadband Luminescence in Rare Earth Doped Sr_2_SiS_4_: Relating Energy Levels of Ce^3+^ and Eu^2+^

**DOI:** 10.3390/ma6083663

**Published:** 2013-08-21

**Authors:** Anthony B. Parmentier, Philippe F. Smet, Dirk Poelman

**Affiliations:** 1 LumiLab, Department of Solid State Sciences, Ghent University, Krijgslaan 281/S1, Ghent 9000, Belgium; E-Mails: anthony.parmentier@ugent.be (A.B.P.); dirk.poelman@ugent.be (D.P.); 2 Center for Nano and Biophotonics (NB Photonics), Ghent University, Ghent 9000, Belgium

**Keywords:** cerium, luminescence, thiosilicate, phosphor, Dorenbos, sulfide, europium, XRD

## Abstract

Sr${}_{2}$SiS${}_{4}$:Ce${}^{3+}$ is an efficient blue-emitting (460 nm) phosphor, excitable with light of wavelengths up to 420 nm. From the excitation spectrum, we construct the energy level scheme and use it to check the predictive power of the Dorenbos model, relating the positions of the Ce${}^{3+}$ energy levels with those of Eu${}^{2+}$ in the same host. For strontium thiosilicate, this method gives excellent results and allows us to determine which of two available crystallographic sites is occupied by cerium. We use the Dorenbos method for extracting information on the coordination of Ce${}^{3+}$ from the observed crystal field splitting.

## 1. Introduction

Sulfides have been used for a very long time as hosts in impurity-doped luminescent materials [1]. The chemical stability of sulfides is rather limited; therefore, some precautions are needed when these materials are used in practical applications. This disadvantage is often dwarfed by the huge advantages exhibited by this material class as a host for luminescent ions, especially rare earth ions. The wide spectrum of colors—especially in the long wavelength range upon doping with divalent europium—that can be obtained, the often broad range of preparation possibilities at relatively low temperature and cost and the high efficiency of some of these phosphors has resulted in there still being a lot of sulfide research being performed [1,2]. This research is mainly aimed at finding new phosphors, improving the efficiency of existing ones and using them for new applications. Recently, protective coatings of luminescent particles were successfully applied to enhance the stability of these sulfide materials [3,4,5]. Particularly vivid are the studies on binary sulfides [6,7,8,9,10,11], thiogallates [12,13,14,15] and thiosilicates [16,17,18,19,20,21,22,23,24,25]. The thiosilicates are, in many practical aspects, comparable to the thiogallates, but considering the depletion of certain natural resources, silicon is to be preferred over gallium, since it is far more abundant and cheaper.

The thiosilicates are good hosts for divalent europium luminescence, but the resulting optical spectra are rather difficult to interpret, because of the complicated set of energy levels of Eu${}^{2+}$, due to the involvement of seven 4f-electrons. Therefore, if one wants to derive information on the influence of the host and the composition on the energy levels, it is more suitable to use Ce${}^{3+}$ as a dopant, because of its simpler electron configuration. Here, we report on Sr${}_{2}$SiS${}_{4}$ doped with Ce${}^{3+}$. This compound was briefly mentioned in the 1970s in a work on cathodoluminescence of alkaline earth thiosilicates, but the luminescence intensity was reported to be weak [26]. After reporting the photoluminescence characteristics of cerium in this material, we also use it as a test case for selected aspects of the recent energy level modeling work of Dorenbos.

A large part of the work of Dorenbos deals with the position of the energy levels of the lanthanides in inorganic materials (For a comprehensive overview: [27]). It is known that these vary in a systematic way when different lanthanides are doped into the same host, much in the same way as the gaseous free ions. Similarly, when a single rare earth ion is used in different hosts, there is also a systematic change in energy levels, related to the nature of the host. As most optical properties depend fundamentally on the position of the energy levels, systematic changes of optical parameters can also be found, when going from one host to another for the same lanthanide or when going from one rare earth to another in the same host.

Rather than working “bottom-up” to solve this problem, by starting from a model for the material and calculating the energy levels of a lanthanide (or other ion) in this material *ab initio* [28,29,30], Dorenbos uses a “top-down” approach that can be summarized as follows: for a very large number of compounds and as many lanthanides as possible, empirical relationships between almost all optical properties (first excitation energy, stokes shift, thermal quenching temperature, …) and all relevant material characteristics (local symmetry of the lanthanide site, chemical nature of ligands, band gap energy, …) are established. These relations are already very useful as such, allowing the prediction or interpretation of the optical characteristics of a certain lanthanide in a certain host. Beyond that, Dorenbos uses these relationships as guidelines to construct detailed physical models that underlie the observed trends.

In the present work, we focus in particular on the relation between the luminescence of Ce${}^{3+}$ in a specific thiosilicate host and the luminescence of Eu${}^{2+}$ in the same host. This aspect of relating the electronic transitions of two different lanthanide ions in the same host is treated by Dorenbos in [31]. We will apply these empirical relations to Sr${}_{2}$SiS${}_{4}$:Ce${}^{3+}$ and show that, in this case, the correspondence between prediction and measurement is excellent.

A second theme that will be explored further is the relation between the optical measurements and the crystal structure, in particular, as treated by Dorenbos in [32].

## 2. Experimental

All samples were prepared by heating SrS (99.9%, Alfa Aesar Inc., Ward Hill, MA, USA), Si (99.9%, Alfa Aesar Inc.) and CeF${}_{3}$ ( 99.9%, Cerac Inc., Milwaukee, WI, USA) in a tube furnace under constant H${}_{2}$S flow. The samples were heated to 850 ${}^{\circ }$C in 3 h and were kept at 850 ${}^{\circ }$C for 1 h. Then, they were allowed to cool down to room temperature. No special measures for charge compensation were taken. Unless specified otherwise, the dopant concentration of the compound under consideration is 0.5%, *i.e*., 0.5% of Sr ions are replaced by the dopant ion. A Bruker D5000 θ−2*θ* diffractometer, using Cu-Kα radiation, was used for X-ray diffraction (XRD) measurements. An FS920 Edinburgh Instruments fluorescence spectrometer was used to collect emission and excitation spectra. Measurements at 10 K were performed by using liquid helium, with an Optistat CF helium cryostat (Oxford Instruments). A Varian Cary 500 UV-VIS-NIR spectrophotometer (Varian Inc., Palo Alto, CA, USA) equipped with an integrating sphere was used to measure the diffuse reflectance of an undoped Sr${}_{2}$SiS${}_{4}$ powder, in order to determine the onset of the host absorption.

## 3. Results and Discussion

### 3.1. Structure

**Figure 1:**
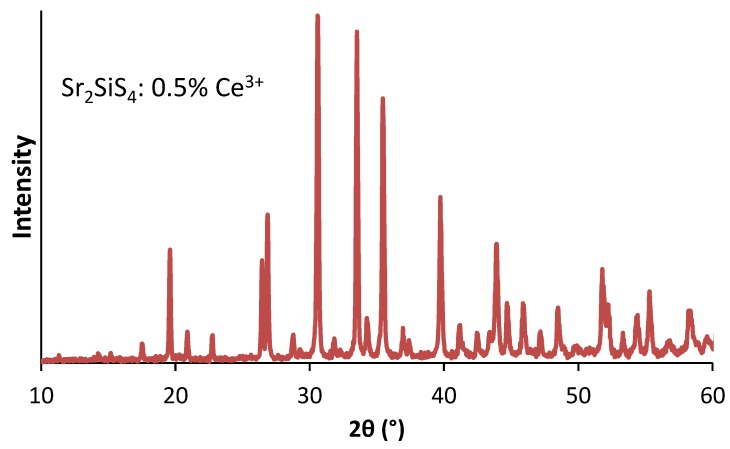
The X-ray diffraction (XRD) pattern of Sr${}_{2}$SiS${}_{4}$:Ce${}^{3+}$ (0.5%).

The space group of Sr${}_{2}$SiS${}_{4}$ was reported to be the monoclinic P2${}_{1}$/m (nr 11) [21]. In Figure 1, the XRD pattern of the synthesized phosphor is plotted.

**Figure 2:**
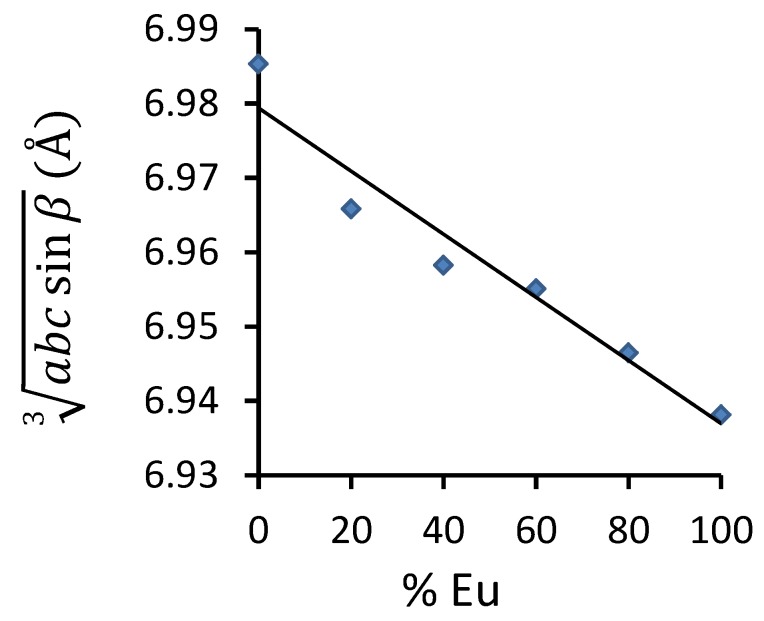
An average lattice parameter (cube root of monoclinic cell volume) as a function of Eu concentration in Eu${}_{2x}$Sr${}_{2-2x}$SiS${}_{4}$. From the refinement results, the error on the lattice parameter is estimated around 0.01 Å. The error on the Eu/Sr ratio is determined by preparation accuracy and is estimated to be on the order of 1%.

Herein, all peaks could be attributed to singe phase Sr${}_{2}$SiS${}_{4}$. This phase is very similar to Eu${}_{2}$SiS${}_{4}$, studied in detail by Hartenbach *et al*. [33]. Therefore, we use the structural parameters of Eu${}_{2}$SiS${}_{4}$ as a starting point for the refinement of Eu${}_{2x}$Sr${}_{2-2x}$SiS${}_{4}$. The lattice parameters decrease quasi-linearly when going from *x* = 0 to *x* = 1, the fully substituted Eu${}_{2}$SiS${}_{4}$. In Figure 2, this is illustrated with a plot of the cube root of the monoclinic cell volume ($\sqrt[{3}]{abc\sin\beta }$).

As expected, when replacing Sr${}^{2+}$ (1.26 Å [34]) by the slightly smaller Eu${}^{2+}$ (1.25 Å [34]), the lattice parameters decrease monotonically, although less than 1%. This difference being small, we use the atomic positions given by Hartenbach *et al*. [33] to evaluate the distances within the crystal. We do correct for the larger lattice parameters of Sr${}_{2}$SiS${}_{4}$, as indicated in Figure 2.

The crystal structure of Sr${}_{2}$SiS${}_{4}$ provides two Sr-sites. To be consistent with the notation of Hartenbach *et al*., for Eu${}_{2}$SiS${}_{4}$, we use the name “Sr1-site” for the analogon of the Eu1-site and “Sr2-site” for the analogon of the Eu2-site [33]. Both are Wyckoff 2e-sites. When divalent europium is used as the dopant, both strontium sites can, in principle, be occupied by the europium ion. This was supported by the occurrence of two emission peaks (480 nm and 550 nm) for europium in this material, with an apparent preference for the Sr1-site. This was derived from the structural and optical behavior of the (Ca,Sr)${}_{2}$SiS${}_{4}$ solid solution [21]. For clarity, we recapitulate this behavior briefly. When Sr${}^{2+}$ is replaced by the smaller Ca${}^{2+}$, only the long wavelength emission peak changes significantly, suggesting that Ca${}^{2+}$ occupies only one of the two sites. The accompanying structural change in lattice parameters is only significant along the c-axis, a dimension mainly determined by the Sr1-site. This suggests that, very probably, the site occupied by Eu${}^{2+}$ in Sr${}_{2}$SiS${}_{4}$ is the Sr1-site, and it is also the long wavelength site. This Sr1-site is the logical candidate to be occupied by Ce${}^{3+}$ (1.143 Å [34]), because everything indicates that it is the site occupied by the similarly sized Ca${}^{2+}$ (1.12 Å [34]) in the situation described above. In Section 3.5, we will present additional support for this hypothesis.

**Figure 3:**
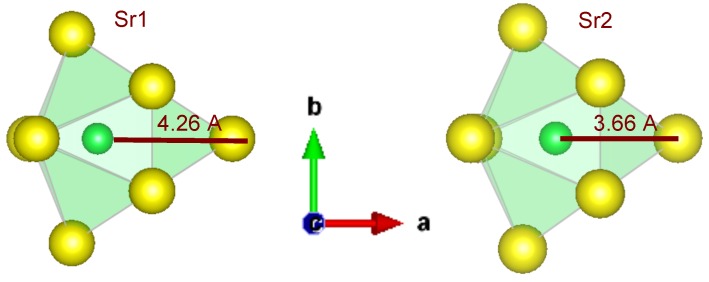
Schematic representation of the coordination environment for both Sr sites in Sr${}_{2}$SiS${}_{4}$.

The Sr1-site is coordinated by six S ions at distances between 3.0 and 3.2 Å, arranged in a distorted trigonal prism. Two of the faces of this prism are capped with a S ion at a distance of around 3.4 Å. The S ion in front of the third face of the prism is at a significantly larger distance, of around 4.3 Å. This is clearly larger than the distance of the Sr1-site to the closest Si, which is around 3.5 Å. Therefore, the net coordination of the Sr1-site is said to be eight-fold or a distorted bi-capped trigonal prism with approximate point symmetry C${}_{2v}$. Both sites are represented in Figure 3, a view from above the capped prism, such that the capping S ions can clearly be seen.

The distances from the Sr ion to the eighth and ninth nearest S${}^{2-}$-ions is also given in Table 1, together with the distances as defined by Dorenbos in [35] and treated in Section 3.4.

**Table 1:** Distance between the Sr and S ions for both Sr sites in Sr${}_{2}$SiS${}_{4}$, and determination of the average distance, R${}_{av}$, in the case of taking coordination number (CN) eight and nine. ΔR is the difference in ionic radius between the replaced and replacing ion. R${}_{eff}$ is the measure of the effective distance after correction for lattice relaxation by introduction of Ce${}^{3+}$. These are the quantities and notations as defined and used by Dorenbos in [35]. All distances are in Å.

Site	Sr1		Sr2	
**CN**	**8**	**9**	**8**	**9**
Sr-S distances	3.02	3.02	3.01	3.01
	3.05	3.05	3.03	3.03
	3.05	3.05	3.03	3.03
	3.08	3.08	3.07	3.07
	3.08	3.08	3.12	3.12
	3.19	3.19	3.12	3.12
	3.43	3.43	3.48	3.48
	3.43	3.43	3.48	3.48
		4.28		3.68
R${}_{av}=<R_{i}>$	3.164	3.289	3.167	3.224
$\Delta R=R_{{\mathrm{Sr}}^{2+}}-R_{{\mathrm{Ce}}^{3+}}$	0.117	0.117	0.117	0.117
R${}_{eff}$ = R${}_{av}-\frac{\Delta R}{2}$	3.11	3.23	3.11	3.17

The Sr2-site is quite similar: six S ions are at distances between 3.0 and 3.2 Å and two capping S ions are around 3.5 Å. However, a third capping S ion is significantly closer—around 3.7 Å—than in the case of the Sr1-site. Therefore, we can do as Hartenbach *et al*. and say that the Sr2-site is 2 + 1 capped, and the coordination is 8 + 1-fold [33]. The symmetry of this lanthanide site can possibly deviate from the bicapped trigonal prism and resemble the tricapped trigonal, D${}_{3h}$. In this context, it is important to note that the silicon ions closest to the Sr2-site are at around the same distance.

### 3.2. Emission Spectrum

**Figure 4:**
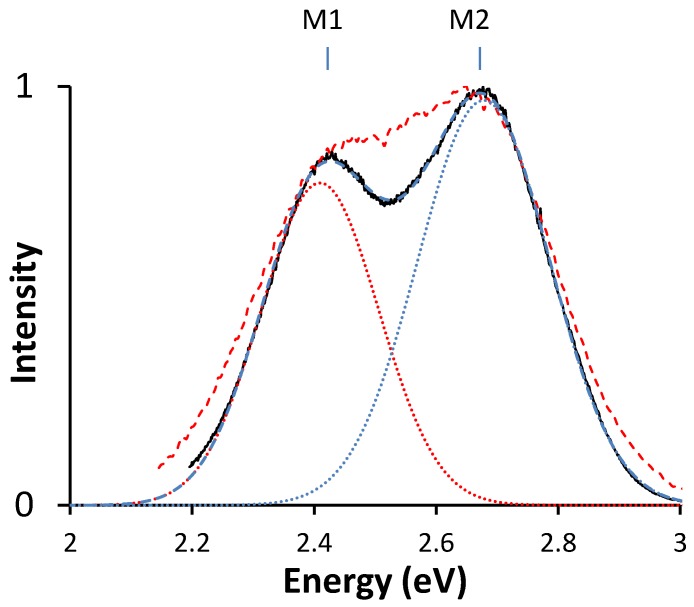
The emission spectrum of Sr${}_{2}$SiS${}_{4}$:Ce${}^{3+}$ at room temperature (red dashed line) and at 10 K (black solid line), both on excitation with 3.88 eV (320 nm). The two Gaussian curves used for fitting are represented with dotted lines. The sum of these curves is the blue dashed line.

The emission spectrum (both at room temperature and 10 K) of Ce${}^{3+}$ in Sr${}_{2}$SiS${}_{4}$ is displayed in Figure 4.

Emission peaks are found at 466 nm (2.66 eV) and 519 nm (2.39 eV). This separation is typical for the spin orbit splitting of the 4f ground level of cerium (0.27 eV or 2153 cm${}^{-1}$, [36]). These values are in line with the measurements reported by Avella. Avella studied the cathodoluminescence of Sr${}_{2}$SiS${}_{4}$:Ce${}^{3+}$ and reported the intensity to be weak [26]. In photoluminescence, however, for sufficiently low concentrations (less than 1%) and excited with light of 400 nm, the phosphor shows a bright emission intensity. The highest luminescence intensity was obtained at a concentration of 0.5 mol percent, under 365 nm excitation. In the XRD spectra of the low dopant concentration samples, no extra peaks are observed. Therefore, we can consider the incorporation in the host quasi-complete, and charge compensation does not seem to be an issue. At higher concentration, concentration quenching sets in, and XRD measurements show that incorporation is not complete (not shown). To improve the performance of the phosphor at higher dopant concentrations, measures for charge compensation should be taken, such as co-doping with a suitable monovalent ion. As the charge compensation does not seem to be a problem at low concentrations, we limit ourselves to this case.

The spectrum shows that only one Sr-site, probably Sr1—based on the structural arguments above and the Dorenbos model below—is substantially occupied by cerium. Indeed, if the second site would also be occupied, we would expect a second pair of emission peaks, shifted toward higher energies. Therefore, we conclude that there is almost no Ce${}^{3+}$ on the Sr2-site or the emission from this site is seriously hampered.

In Section 3.5, evaluating the empirical relation between Ce and Eu will support the hypothesis that the cerium ion occupies the same site that is preferentially occupied by the Eu${}^{2+}$ ion.

### 3.3. Excitation Spectrum

**Figure 5:**
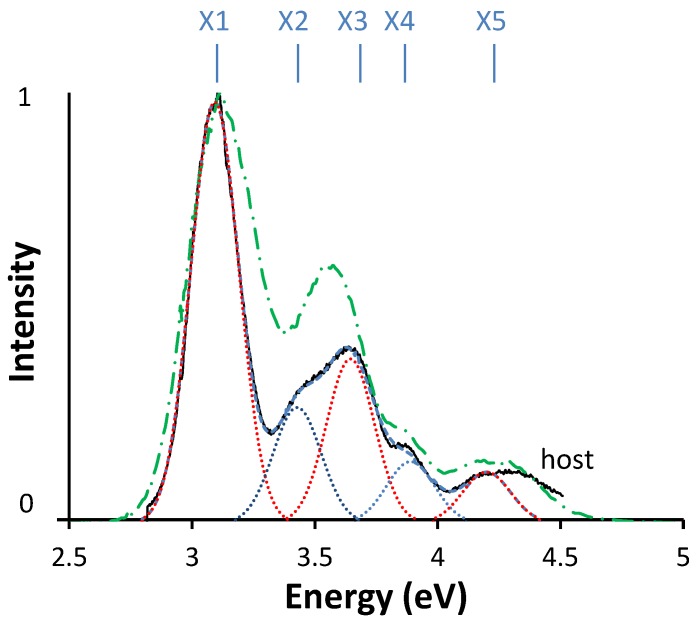
Excitation spectrum of Sr${}_{2}$SiS${}_{4}$:Ce${}^{3+}$ at room temperature (green dashed line) and at 10 K (black solid line), when monitoring the emission at 2.25 eV (550 nm). A fitting curve is also shown for 10 K. The individual Gaussian curves (all with equal width) for all five peaks (X1, …, X5) are shown as dotted lines.

In Figure 5, the excitation spectrum of Ce${}^{3+}$-doped Sr${}_{2}$SiS${}_{4}$ is shown.

At least four distinct peaks can be discerned in the spectrum measured at room temperature. To confirm if (and which) one of the peaks is composed of more components, a measurement at 10 K was performed. This revealed that the second peak, found to be around 3.65 eV at room temperature, consists of two bands, at 3.43 eV and 3.64 eV. The peak on the high energy end of the excitation spectrum is relatively broad and also seems to be complex. In order to clarify this, a diffuse reflection measurement was carried out (spectrum not shown). The onset of the host absorption is found around 4.35 eV (285 nm). Therefore, we attribute the high energy part of this peak to host absorption.

In total, the position of all 5d peaks can be determined with reasonable accuracy. All relevant numbers are compiled in Table 2.

In the next section, we use these values to construct an energy level scheme for Sr${}_{2}$SiS${}_{4}$:Ce${}^{3+}$.

**Table 2:** Table with measured and calculated values for Sr${}_{2}$SiS${}_{4}$:Ce${}^{3+}$ and Sr${}_{2}$SiS${}_{4}$:Eu${}^{2+}$. D(n+) is the redshift of the lowest 5d excitation level as compared to that of the free n-valent ion; $\varepsilon {}_{\mathrm{CFS}}$ is the crystal field splitting of the 5d manifold; Δ is the spin orbit splitting of the cerium ground state; $\varepsilon {}_{c}$ is the centroid shift of the 5d centroid; $\varepsilon {}_{s}$ is the crystal field shift; and ΔS is the stokes shift between first excitation and the emission band.

Measured Ce${}^{3+}$	eV	Calculated, Ce${}^{3+}$	eV	cm${}^{-1}$	Estimated, Eu${}^{2+}$	eV	cm${}^{-1}$
X1	3.09				X1,Eu	2.51	
X2	3.43						
X3	3.64	< X >	3.65				
X4	3.89						
X5	4.20						
		D(3+)	3.03	24,400	D(2+)	1.71	13,800
		$\varepsilon {}_{\mathrm{CFS}}\left({\mathrm{Ce}}^{3+}\right)$	1.11	8,950	$\varepsilon {}_{\mathrm{CFS}}\left({\mathrm{Eu}}^{2+}\right)$	0.855	6,890
		$\varepsilon {}_{c}\left({\mathrm{Ce}}^{3+}\right)$	2.70	21,800			
		$\varepsilon {}_{s}\left({\mathrm{Ce}}^{3+}\right)$	0.56	4,500			
M1	2.41	Δ	0.27	2,200	M,Eu	2.25	
M2	2.68						
		ΔS(3+)	0.41	3,300	ΔS(2+)	0.25	2,000

### 3.4. Energy Level Scheme

**Figure 6:**
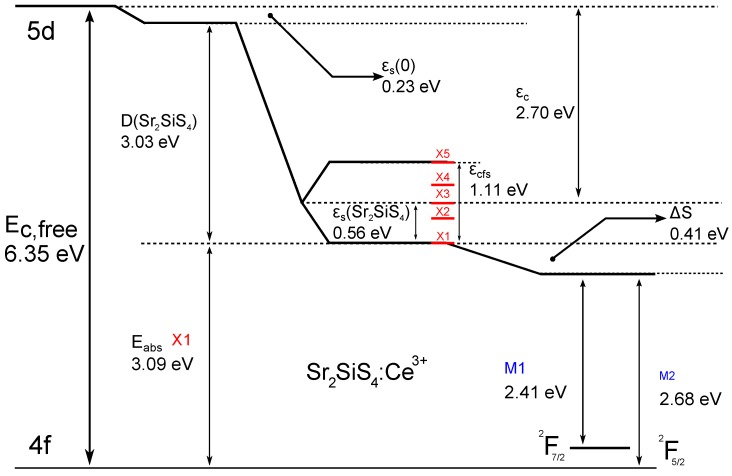
Energy level scheme for Ce${}^{3+}$ in Sr${}_{2}$SiS${}_{4}$:Ce${}^{3+}$.

The cerium 5d levels of the free ion, at 6.35 eV above the 4f ground state [36], shift and split when the ion is incorporated in a crystal with low symmetry. In the excitation spectrum presented in the previous section, we see the degeneracy being lifted. The resulting energy level scheme is given in Figure 6.

The type of splitting (number of resolved peaks) is a function of the site symmetry. Five peaks are needed to fit the spectrum, and it is useful to check if this is in line with expectations.

We can do that by using the trends observed by Dorenbos in [32], where a relation between the crystal field splitting and the type of anion coordination polyhedron was established. Using the average distance from ion to the ligands (R${}_{av}$ = 3.16 Å) and the correction for lattice relaxation when doping the smaller cerium in the larger strontium site (ΔR=1.26−1.143 Å), we find R${}_{eff}$ = R${}_{av}$−$\frac{\Delta R}{2}$ = 3.11 Å if we suppose that the Sr-site is eight-coordinated. With this value, the (R${}_{eff}$,$\varepsilon {}_{\mathrm{CFS}}$)-combination fits the trend for eight-coordinated polyhedrons far better than the trend for a tricapped trigonal prism (CN = 9) polyhedra [32]. Indeed, if we repeat the calculation with the first nine neighbors, the (R${}_{eff}$,$\varepsilon {}_{\mathrm{CFS}}$)-combination does not fit the Dorenbos trend for nine-coordinated polyhedrons at all. Within the accuracy that can be expected from this reasoning, this supports the point of view that the site is eight-coordinated, rather than nine-coordinated to S ions and, therefore, that the site occupied by Ce${}^{3+}$ is the Sr1-site.

The accuracy of this analysis is, however, limited by several factors:

- The position of the excitation peak with the highest energy is obscured by host absorption. This lowers the accuracy of the value for the highest 5d level position and, thus, for the value of the crystal field splitting.
- The average distance between dopant and ligand is, of course, determined by the choice of the number of S ions that are considered nearest neighbors. As discussed in the section on structure, eight or nine S ions could be considered nearest neighbors. In any case, the result is closer to the trend of eight-coordination than to that of nine-coordination.

In support of the value (2.70 eV) provided for the centroid shift of Sr${}_{2}$SiS${}_{4}$:Ce${}^{3+}$, we note that it is comparable with the values for the ternary sulfides, quoted in [37], e.g., SrGa${}_{2}$S${}_{4}$: 2.61 eV ; CaGa${}_{2}$S${}_{4}$: 2.75 eV.

### 3.5. Eu${}^{2+}$ Characteristics from Ce${}^{3+}$

Using Dorenbos’ model from [31], there are two ways to predict the europium characteristics from the cerium energy levels. The difference between the first 5d absorption energy for gaseous Ce${}^{3+}$ and the same ion doped in Sr${}_{2}$SiS${}_{4}$ is called the redshift D(Sr${}_{2}$SiS${}_{4}$,3+). On the first order, this value is the same for all trivalent lanthanides in Sr${}_{2}$SiS${}_{4}$, if on the same site.

We can first use the following empirical relationship to find the redshift for divalent lanthanides from the redshift of trivalent lanthanides.

(1) D(2+)=0.64×D(3+)−0.233eV

In that way, using the Eu${}^{2+}$ free ion value and the calculated redshift D(2+), we find 2.49 eV (498 nm) for the first excitation energy of Eu${}^{2+}$ in the same host. In order to also estimate the emission energy via Formula 1, we can use the empirical formula that relates the stokes shift (ΔS(3+)) of the trivalent lanthanides with that of the divalent ones (ΔS(2+)). Indeed, on the first order, this stokes shift is also approximately the same for all lanthanides in the same host.

(2) ΔS(2+)=0.61×ΔS(3+)

Using these relations, we calculate the emission energies in Sr${}_{2}$SiS${}_{4}$ doped with europium: 2.25 eV (551 nm). In [21], we reported an emission peak of around 2.23 eV (556 nm) at 10 K, a value in line with the prediction above. The first excitation peak for europium is more difficult to obtain from these experimental spectra, but a good estimate is between 480 nm and 500 nm, also in agreement with the value above.

We can check the redshift against other known values, by noting that it should be more or less constant for all divalent lanthanides in the same compound. Possible candidates are ytterbium, samarium or thulium. Only for ytterbium, we found broadband emission, and we determined the first 5d excitation level of Yb${}^{2+}$ in Sr${}_{2}$SiS${}_{4}$ to be around 2.6 eV. Using the free ion value of ytterbium ([36]), we find a redshift value for the divalent lanthanides of 1.54 eV. This is in the same range as the calculated value of 1.71 eV, considering that the redshift for ytterbium is often less than for europium [38]. We will treat the case of ytterbium in thiosilicates in detail in later work.

A second way to calculate the optical properties of divalent europium, starting from the measurements on trivalent cerium, is proposed by Dorenbos and implies using the empirical formula for the (excitation or emission) transition energies:

(3) $$E\left({\mathrm{Eu}}^{2+}\right)=0.64\times E\left({\mathrm{Ce}}^{3+}\right)+0.53\;\mathrm{eV}$$

This results in values of 2.51 eV (494 nm) for the excitation band with the lowest energy and 2.24 eV (554 nm) for the emission energy. All values are listed in Table 2, with the results for cerium in the first column and the calculated values for europium in the second column.

Both values are in line with the expected values, provided that the Ce${}^{3+}$ ion occupies the Sr1-site. We consider this as a confirmation of the hypothesis that both europium and cerium occupy the Sr1-site preferentially.

Dorenbos also provides the following empirical relationship for crystal field splitting.

(4) $$\varepsilon {}_{\mathrm{CFS}}\left(\mathrm{Eu}\right)=0.77\times \varepsilon {}_{\mathrm{CFS}}\left(\mathrm{Ce}\right)$$

It is used to estimate the value provided in Table 2 for the crystal field splitting of the 5d level in Eu${}^{2+}$-doped Sr${}_{2}$SiS${}_{4}$. This value for the crystal field splitting for europium (from around 360 nm to 495 nm) is in general agreement with the excitation spectrum presented in [21], Figure 6.

## 4. Conclusions

Summarizing, we presented the luminescent characteristics of Sr${}_{2}$SiS${}_{4}$:Ce${}^{3+}$. From the excitation spectrum, we distilled an energy level scheme, with reasonably accurate values for centroid shift, crystal field splitting and redshift. We used the empirical Dorenbos relation between Eu${}^{2+}$ and Ce${}^{3+}$ luminescence to show that the Ce${}^{3+}$ ion occupies the same site that is occupied preferentially by europium in the same host. By using Dorenbos’ model for the relation between the cerium crystal field splitting and its coordination polyhedron type, we showed that this Ce${}^{3+}$ behaves more as eight-coordinated than as nine-coordinated. In this way, we conclude that in Sr${}_{2}$SiS${}_{4}$, both Eu${}^{2+}$ and Ce${}^{3+}$ preferentially occupy the eight-coordinated Sr1-site.
